# Malignant features of minipig melanomas prior to spontaneous regression

**DOI:** 10.1038/s41598-024-59741-w

**Published:** 2024-04-22

**Authors:** Héloïse Débare, Fany Blanc, Guillaume Piton, Jean-Jacques Leplat, Silvia Vincent-Naulleau, Julie Rivière, Marthe Vilotte, Sylvain Marthey, Jérôme Lecardonnel, Jean-Luc Coville, Jordi Estellé, Andrea Rau, Emmanuelle Bourneuf, Giorgia Egidy

**Affiliations:** 1 Université Paris-Saclay, INRAE, AgroParisTech, GABI, 78350 Jouy-en-Josas, France; 2 Université Paris-Saclay, CEA, Stabilité Génétique Cellules Souches Et Radiations, 92260 Fontenay-Aux-Roses, France; 3 Université de Paris Cité, CEA, Stabilité Génétique Cellules Souches Et Radiations, 92260 Fontenay-Aux-Roses, France; 4 Université Paris-Saclay, INRAE, AgroParisTech, Institut Micalis, 78350 Jouy-en-Josas, France

**Keywords:** Cancer models, Oncogenes, Skin cancer, Tumour biomarkers, Tumour immunology

## Abstract

In MeLiM minipigs, melanomas develop around birth, can metastasize, and have histopathologic characteristics similar to humans. Interestingly, MeLiM melanomas eventually regress. This favorable outcome raises the question of their malignancy, which we investigated. We clinically followed tens of tumors from onset to first signs of regression. Transcriptome analysis revealed an enrichment of all cancer hallmarks in melanomas, although no activating or suppressing somatic mutation were found in common driver genes. Analysis of tumor cell genomes revealed high mutation rates without UV signature. Canonical proliferative, survival and angiogenic pathways were detected in MeLiM tumor cells all along progression stages. Functionally, we show that MeLiM melanoma cells are capable to grow in immunocompromised mice, with serial passages and for a longer time than in MeLiM pigs. Pigs set in place an immune response during progression with dense infiltration by myeloid cells while melanoma cells are deficient in B2M expression. To conclude, our data on MeLiM melanomas reveal several malignancy characteristics. The combination of these features with the successful spontaneous regression of these tumors make it an outstanding model to study an efficient anti-tumor immune response.

## Introduction

Malignancy is the tendency of cancer to invade and destroy nearby tissue, metastasize, and cause death. There is a widely accepted set of capabilities that normal cells must acquire in order to become malignant. In this sense, the hallmarks of cancer highlight features that render proliferative cells autonomous and checkpoint insensitive, with the ability to evade immune destruction, reprogram cellular metabolism and take advantage of genome instability and tumor-promoting inflammation^1^. The cancer hallmarks have been associated with Gene Ontology (GO) biological processes resources^2–6^.

Melanoma is the deadliest form of skin cancer, and its incidence is increasing, particularly in young adults. Its 5-year survival rate for patients with metastatic disease still does not exceed 30%^7^. In addition to humans, melanoma can occur in almost all pigmented animals^8^. The pig is a well-established biomedical model used for skin physiological studies for decades^9^. In pigs, melanocytes are located in the basal layer of the epidermis and dark-coated pigs have a high incidence of melanoma. In the MeLiM breed, cutaneous melanomas develop during the perinatal period. The histopathologic characteristics of MeLiM melanomas are similar to those in humans^10^. Nodular cutaneous lesions are associated with metastatic disease. MeLiM minipigs can develop concurrent primary melanomas at multiple sites. In humans, the outcome of patients with multiple primary cutaneous melanoma has been the subject of conflicting reports^11–13^. In both the original Czech MeLiM herd^14^ and the French herd^15^, genetic analysis has shown that melanoma incidence and progression are caused by genetic factors with minimal environmental influence^16^. While common genes known to induce melanoma in humans are not mutated in the MeLiM genome, causal genes for this tumor model remain to be identified. Cellular transformation in the MeLiM model occurs in the absence of generalized constitutive melanocytic hyperplasia, which is commonly described in genetically engineered melanoma mouse models such as *Braf*^*V600E*^ ^17^ or *Nras*^*Q61K*^ ^18^. The MeLiM model may therefore be a particularly relevant model for human melanoma development, surpassing mouse models in some aspects.

Indeed, another fascinating feature of this model is that, intriguingly, MeLiM melanomas regress from the second month of age. Lesions dry out and depigment, and animals eventually survive, even when lymph node metastases have been clinically detected^10^. Depigmentation can extend to the entire animal coat and its severity has been associated with a specific *CD4* haplotype^19^. Longitudinal transcriptional analysis showed that regression is likely due to a combination of immune response and cell-autonomous mechanisms^20^. The immune cell subsets involved in this regression process has recently been characterized emphasizing the dynamic accumulation of different macrophage subtypes and the later infiltration of lymphoid cells^21^. While in the original Czech herd 34% of melanoma-affected minipigs died prematurely^14^, in the French herd only 5.4% died^10^; therefore, as most MeLiM recover successfully from melanoma, questions remain about the malignancy of these tumors.

However, advances in immuno-oncology over the past decade have challenged many paradigms, demonstrating the early establishment of an anti-tumor response and the ability to control tumor progression in late-stage melanoma by breaking immune tolerance^22^. Concomitantly, the mitogen-activated protein kinase (MAPK) and phosphatidylinositol 3-kinase (PI3K)/protein kinase B (AKT) pathways have been shown to be activated in most melanomas. Dysregulation of these pathways is often due to mutations in the *B-RAF, NRAS, NF1, KIT, PTEN* or *AKT3* genes, the rates of which have led to a genomic classification of melanomas^23^. While immunotherapies have been shown to induce durable responses in a small percentage of patients, targeted therapies using MAPK inhibitors have rapidly but transiently improved median survival despite their immunomodulatory effects^24^. Combined therapies have shown that both intrinsic cancer cell characteristics and components of the tumor microenvironment are critical to cancer outcome^25^.

The MeLiM minipig model could help address the unmet need to understand the pro and anti-tumor roles of the immune system. To consider whether MeLiM mechanisms of regression would be of interest for translation to human medicine, it is crucial to determine whether MeLiM melanoma cells are overtly malignant. To this end, we retrospectively analyzed nascent, early age lesions at different time points during tumor progression, prior to clinical signs of regression, examined their transcriptome and mapped differentially expressed genes to GO processes associated with hallmarks of cancer. We also analyzed genome instability, proliferation marks, and activation of kinases reported to be necessary for human melanoma growth, and sequenced significantly mutated genes in human melanoma^23,26^. As a functional assay we performed serial in vivo engraftment experiments that can distinguish between hyperplastic and malignant growth^27^. We show here that in the MeLiM model, cure is not incompatible with malignant features.

## Results and discussion

### MeLiM melanoma clinical and histologic features

To characterize clinical and histologic tumor progression, we sampled 70 growing tumors of nodular type present at birth, collected on 60 piglets aged 8–49 days. None displayed clinical signs of regression, i.e. drying surface, flattening and depigmentation of the tumor, despite the histologic signs of regression that were later noticed among less early lesions. Tumor burden was mostly medium or high with concurrent multiple primary melanomas. All pigs survived melanoma with complete remission. Aiming at identifying malignancy traits, we defined three groups of lesions according to animal age at sampling: (1) the EP group (early progressive lesions) was sampled between 3 and 12 days of age; later samples (13–49 days) were distinguished by the histologic presence of fibrosis in HES staining of lesions such that (2) the P group (progressive lesions) comprised lesions between 13 and 42 days without fibrosis; and (3) the PF group (progressive with fibrosis) enclosed lesions sampled at 19–49 days (Fig. S1). Individual data on pigs and lesions are presented in Tables S1 and S2, respectively, and comparisons of the EP, P and PF groups are summarized in Table 1. Human melanoma malignancy is staged according to thickness, ulceration and metastasis. As for clinical traits of malignancy, MeLiM lesions became gradually larger over time (PF group lesions were larger in size although they evolved more slowly) and were more associated with palpable hard adenomegaly, a proxy of the metastasis process (76.2% in PF versus 24.2% in P versus 6.3% in EP). Ulceration was common in tumors of all groups, with a tendency to decrease over time (Table 1). As for histologic traits of malignancy, tumor growth was mainly vertical and increased with time (PF had a significantly greater depth compared to other groups), and intralesional lymphocytic infiltration was higher in the PF group. Histologic ulceration was detected in all groups (Table 1). Overall, lesions that initially developed as highly proliferative continued to progress in depth, despite apparent clinical stabilization. Ulceration was frequently seen and in a majority of animals a metastatic process gradually developed. A lymphocytic infiltration, first perilesional and then intralesional, in parallel with increasing fibrosis, suggests an evolution of the tumor microenvironment associated with regression in a clinical malignant frame. The main phenotype of MeLiM melanomas is nodular ulcerating fast-growing tumors often reaching 2 cm in 2-week-old piglets with subsequent adenomegaly and lymphadenopathy. Interestingly, the prevalence of concurrent multiple primary melanomas in our model may also be related to the strong antigenicity of MeLiM lesions. Indeed, studies showing that multiple primary melanomas with metastases are associated with a better outcome in humans suggest that the priming of the immune system by multiple clones of more than one primary tumor may provide a broader host immune response^11^. However, the median overall survival for patients with multiple primary melanomas was only 11.2 months^11^.

**Table 1:** Clinical and histologic characteristics of melanoma progression in MeLiM pigs. Pig lesions were retrospectively classified in EP (early progression), P (progression) and PF (progression with signs of fibrosis) groups. Genotype at the *CD4* locus may be *Ref* (reference sequence) or *Alt* (alternative sequence)^19^.

	EP (n = 16)	P (n = 33)	PF (n = 21)	chi-square or ANOVA *p-*value	Tukey's post Test		
					EP vs P	EP vs PF	P vs PF
Group description							
Pig's age (mean [min–max])	8.9 [3–12]	20.9 [13–43]	34.5 [19–49]				
Presence of fibrosis = partial regression	No	No	Yes				
Sex (M; F)	7; 9	14; 19	9; 12	0.996			
Tumor burden (Low; Medium; High)	0; 6; 10	3; 12; 16	1; 9; 11	0.722			
CD4 (Ref/Ref; Ref/Alt; Alt/Alt)	2; 10; 4	12; 15; 6	7; 12; 2	0.385			
Clinical observations of animals at sampling							
Palpable adenomegalies (%)	**6.3**	**24.2**	**76.2**	**< 0.001**			
Clinical observations of lesions							
Lesion's size (mean in cm ± SD)	**2.13 **± **0.41**	**2.45 **± **0.67**	**3.04 **± **0.83**	**< 0.001**	ns	***	**
Lesion's size evolution (% variation/day ± SD)	**6.67 **± **7.06**	**4.17 **± **2.32**	**1.50 **± **1.77**	**< 0.001**	ns	***	*
Lesion's size evolution				**0.019**			
Increasing (fast) (%)	50.0	51.5	9.5				
Increasing (slow) (%)	43.8	36.4	61.9				
Stable (%)	6.3	12.1	28.6				
Description				**0.040**			
Plateau (%)	6.3	30.3	19.0				
Dome (%)	75.0	60.6	42.9				
Exophytic (%)	18.8	9.1	38.1				
Ulceration				0.083			
No (%)	0.0	30.3	19.0				
Yes (+) (%)	31.3	24.2	42.9				
Yes (++) (%)	68.8	45.5	38.1				
Histological observations of the tumoral zone							
Profile				0.404			
Dome or plateau (%)	64.3	57.6	42.9				
Polypoid (%)	35.7	42.4	57.1				
Clark's level				0.989			
IV (%)	23.1	23.3	25.0				
V (%)	76.9	76.7	75.0				
Ulceration				0.727			
No (%)	15.4	16.1	9.5				
Low (%)	46.2	32.3	28.6				
High (%)	35.7	51.6	61.9				
High vascularization (%)	78.6	93.5	90.5	0.314			
Lymphoid infiltration				0.074			
No (%)	50.0	53.1	42.9				
Yes (perilesional) (%)	50.0	43.8	33.3				
Yes (perilesional & intralesional) (%)	0.0	3.1	23.8				
Lesion's section size							
n	7	19	17				
Length (mean in cm ± SD)	1.42 ± 0.52	1.81 ± 0.77	2.10 ± 0.76	0.122			
Depth (mean in cm ± SD)	**0.63** ± **0.20**	**0.73** ± **0.30**	**1.11** ± **0.33**	**< 0.001**	ns	**	**
Area (mean in mm^2^ ± SD)	**75.2** ± **53.8**	**110.2** ± **80.3**	**173.5** ± **91.2**	**0.017**	ns	*	ns

Significant values are in bold.

### MeLiM melanomas express hallmarks of cancer

In order to explore MeLiM melanoma malignant processes, RNAseq was performed in 12 lesions, 4 lesions at each evolution stage (EP, P and PF; a MDS representation of RNAseq data is provided in Fig. S2). We then defined genes expressed at each stage using a threshold (TPM > 1 for at least 3 out of 4 lesions): 11,311, 11,667 and 12,267 genes were considered as expressed in the EP, P and PF groups, respectively. Most genes (91.1%) were expressed in > 2 groups and only 181, 286 and 681 were uniquely expressed in one of the EP, P and PF groups, respectively (Fig. S3a). The differential expression (DE) analysis performed between groups found 61 DE genes between EP and P groups, 54 EP *vs* PF and 32 P *vs* PF (FDR < 0.05, Table S3). Feature Set Enrichment Analysis (FSEA) was then performed to assess GO terms (category biological process) enriched in sets of expressed genes in each group. EP, P and PF groups displayed 1164, 1118 and 1113 enriched GO terms, respectively. 87.1% of GO terms were found enriched in at least 2 groups and only 92, 43 and 30 were uniquely enriched in EP, P and PF groups, respectively (Fig. S3b). The full lists of GO terms enriched in the groups are provided in Table S4. When applied to DE genes, FSEA revealed 168, 469 and 28 enriched GO terms when comparing EP vs P, EP vs PF and P vs PF groups, respectively (Table S5). While most of the GO terms are related to the immune response and inflammation, with a higher statistical significance in the comparisons between EP lesions and the others, the comparison of P and PF lesions showed weaker differences, including cytotoxicity, angiogenesis or Ag-MHC assembly (Fig. S3c,d).

**Figure 1:**
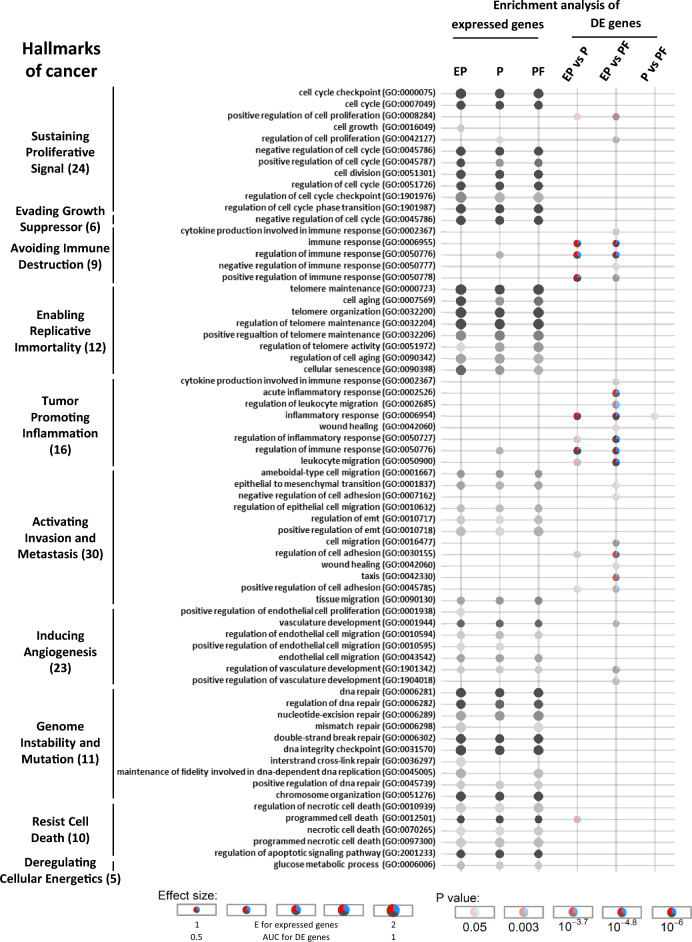
MeLiM early melanomas show all hallmarks of cancer. Longitudinal transcriptomic analysis and FSEA in GO terms of genes expressed in EP, P and PF groups and in DE genes between groups. Hypergeometric tests were used to determine the enrichment of expressed genes in each group (foreground set) among the total RNAseq gene set (background set corresponding to 16,559 genes). The CERNO method was used to analyze gene enrichment in ranked lists of *p*-values from DE analyses. Only GO terms assigned to cancer hallmarks, extracted from Chen et al. ^6^ were plotted and ranked by associated hallmarks (left). The total number of GO terms assigned to each cancer hallmark is shown in parentheses. The strength of the *p*-value is indicated by the transparency of color; the effect size (E or AUC) is indicated by the plot size. Significantly (*p* < 0.05) up-regulated genes in EP tumors are colored red while downregulated genes are colored blue; others are colored gray.

We compared GO enrichment lists among expressed or DE genes to a list of 146 GO terms which were assigned to hallmarks of cancer^6^ to specifically highlight processes involved in cancer cell development and tumorigenesis. These GO term hallmarks of cancer were grouped into ten broad categories^1^, each containing a varying proportion of terms with significant enrichment among expressed genes in at least one of the evolution stage groups or among DE genes between groups (Fig. 1): “genome instability and mutation” with 10 GO terms out of 11, “enabling replicative immortality” with 8/12, “avoiding immune destruction” with 5/9, “resist cell death” with 5/10, “tumor promoting inflammation” with 8/16, “sustaining proliferative signal” with 11/24, “activating invasion and metastasis” with 12/30, “inducing angiogenesis” with 7/23, “deregulating cellular energetic” with 1/5 and “evading growth suppressor” with 1/6. GO terms assigned to “avoiding immune destruction” and “tumor promoting inflammation” are those with the strongest evolution between EP and later stages. The “activating invasion and metastasis” and “inducing angiogenesis” hallmarks were also modulated, but primarily between EP and PF groups.

Thus, MeLiM melanomas transcriptomic profiles of lesions grouped by clinical parameters were mapped to cancer hallmarks using GO terms collected by Chen et al*.*^6^. Although the list of GO terms is not exclusive to cancer and they may evolve, this approach highlights the enrichment of all cancer hallmarks in MeLiM melanomas from early onset, with slight modulation during progression except for immune-related hallmarks which varied significantly. To confirm this, we next focused on the hallmarks best represented in expressed genes and on those with more significant differences during lesion evolution.

### Genome instability in MeLiM tumors with high mutation rates but no UV signature

**Figure 2:**
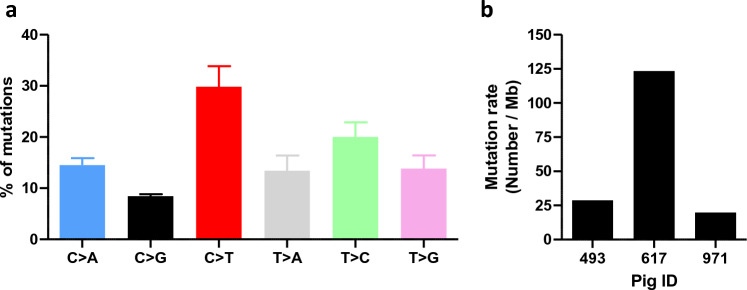
Mutational profile of MeLiM tumors. (**a**) % of mutations found in 19 tumors from 3 pigs according to substitution subtypes, (**b**) tumor mutation rate for each of the 3 pigs.

The cancer hallmark with the higher percentage of GO terms expressed in MeLiM melanomas corresponded to “genome instability and mutation”, with no significant evolution during the time points analyzed. Therefore, we decided to explore tumor genome sequencing data to identify passenger somatic mutations. The patterns of such passenger mutations, or mutational signatures, can reveal molecular processes underlying tumorigenesis, such as external factors^28^. We used data from a custom multi-region exome-based sequencing project to gain insight into the mutational landscape and burden in MeLiM tumors (n = 19 tumors from 3 pigs). Although not exhaustive and only focused on 6.56Mb of the MeLiM genome, this approach revealed pertinent information. We therefore compared the genome sequence from each tumor to the normal DNA counterpart, extracted from the same animal’s blood. First, no recurrent mutations were found across different tumors of the same individual. Although some mutations were located in the same genes, none represented driver genes found in different animals. No common mutation was found between individuals either. Next, we compared the tumor genomes to summarize the 6 different substitution types in tumors from the 3 MeLiM pigs. The mutational spectra are similar between the three individuals, and correspond to a higher load of C>T substitutions, followed by T>C (Fig. 2a). Overall, among the mutational signatures inventoried in COSMIC, the MeLiM tumor signature could correspond to SBS44 (Single Base Substitutions^28^). This signature is not associated to a specific etiology related to external factors such as UV or pollutants. It is rather defined by a « Defective DNA mismatch repair », meaning occurrence of spontaneous nucleotide substitutions and mismatches in the frame of cancer cell growth^29^. The mutational burden was variable between samples, ranging from 20 to 123 Single Nucleotide Variants (SNV)/Mb (Fig. 2b). These data point to a high mutation burden in MeLiM melanomas, which is associated to immunogenicity in human malignant cancers.

The somatic mutation burden in MeLiM melanomas was found to be higher than the average found in human cancers but comparable to that of human melanomas^30^. Human cutaneous melanomas often display a mutational signature specific to UV light exposure^31,32^. Not surprisingly, MeLiM tumors instead have a mismatch repair deficiency (MMR deficiency) signature, which is not specific to any one etiology, but is described as an endogenous process. Interestingly, despite a high mutational burden^33^, half of the tumors associated with an MMR deficiency pattern in large scale human studies fail to identify a driver mutation^28^. The mutational burden found in MeLiM lesions ranges from 20 to 123 SNVs/Mb, which corresponds to a hypermutability phenotype^34^. This is consistent with observations showing that MMR-deficient tumors respond better to anti-PD-1 treatment^35^. The large number of possible neoantigens generated by the mutational load of the tumors may lead to a better response to immune checkpoint inhibitors and ultimately benefit the patient. All of these observations are consistent with MeLiM tumors, which do not carry mutations in the canonical melanoma oncogenes/tumor suppressor genes but still have a high mutational burden and an MMR deficiency signature.

### High tumor cell proliferation rate in MeLIM melanomas

**Figure 3:**
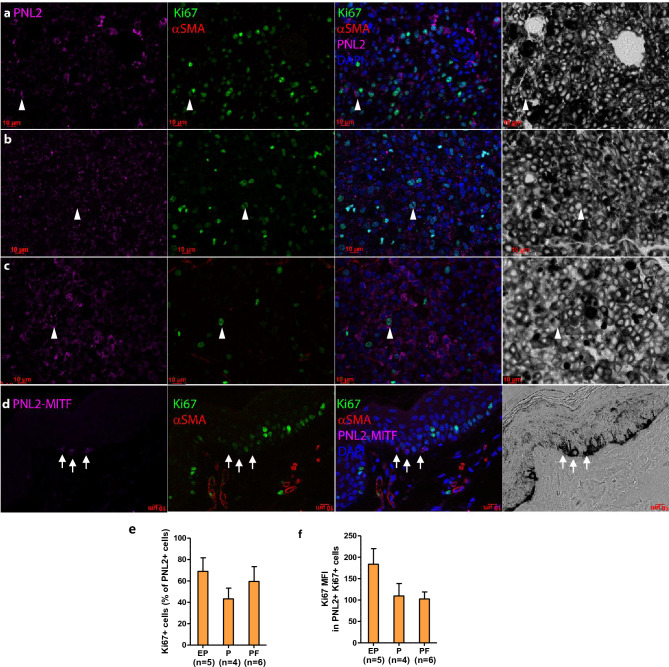
Immunofluorescence analysis of proliferation marks in MeLiM melanoma. Representative regions of EP (**a**), P (**b**), PF (**c**) and control skin of a 22-day-old pig (**d**) with staining for the melanocytic marker PNL2 (and MITF in control skin) (purple), proliferation marker Ki67 (green) and pericyte marker αSMA (red). Third column: merge of the previous with DAPI for nuclei (blue). Last column: corresponding brightfield with high melanin content. PNL2 and αSMA signal is cytoplasmic, Ki67 and MITF signal is nuclear. Arrowheads point to one representative PNL2^+^ Ki67^+^ cell per field. Arrows point to 3 PNL2^+^ Ki67^-^ cells in normal skin. Scale bars 10 µm. (**e**) Quantification of Ki67^+^ cells among PNL2 ^+^ cells. (**f**) Ki67 median fluorescence intensity (MFI) in Ki67^+^ PNL2^+^ cells.

Because of tumor growth speed and the score of sustained proliferative signals of MeLiM melanomas, we characterized the proliferation biomarker Ki67 expression on normal skin and melanoma fixed samples by triple immunofluorescence together with the melanocytic marker PNL2 and the angiogenic marker αSMA. Highly abundant nuclear Ki67^+^ proliferative marks were present in tumor melanocytic cells which appear densely packed (arrowheads in Fig. 3a–c). In skin adjacent to the tumor, only basal keratinocytes were Ki67 positive and PNL2^+^ cells were negative (arrows in Fig. 3d), in sharp contrast to skin above the lesion where Ki67^+^ melanocytic (PNL2^+^, MITF^+^) cells were detected (arrowheads in Fig. S4). Using automated count, the score of Ki67^+^ melanoma cells averaged 68.9 ± 12.7, 43.2 ± 10.0 and 59.5 ± 13.8% in EP, P and PF groups, respectively (Fig. 3e). As signal could be mild or strong, we made use of quantitative data and plotted the distribution of intensities in the field showing a substantial progressive reduction of signal intensity with chronology even though not reaching statistical significance (Fig. 3f). The Ki67 protein, which is expressed at all cell cycle stages, independent of DNA damage repair mechanisms, has been shown to be a more sensitive marker of cell proliferation than mitoses^36^. MeLiM melanoma cells showed 70% of cells Ki67^+^, which is higher than what is regularly reported in human melanoma, with some exceptions such as in anorectal melanoma^37^. Interestingly, the assessment of Ki67 in breast tumors using digital image analysis was higher than visual assessment of the same tumors^38^. A recent meta-analysis reported that high level of Ki67 expression was associated with poor prognosis in melanoma patients, regardless of region and cut-off of Ki67%^39^. Our longitudinal data show that Ki67 signal intensity decreases with progression, which does not appear to be a methodological artefact. Despite a better understanding of the role of Ki67^40^, no studies were found that addressed the variability of Ki67 intensity, although the lowest discordance in Ki67 scoring is achieved when all labeling intensities are counted as positive^41^. This issue requires further investigation.

### Cancer-related pathways are activated in MeLIM melanomas

We sequenced the open reading frames of the *BRAF, NRAS, KRAS, NF1, PTEN* genes known to be drivers of human melanoma^23^ in three animals and compared them to the reference sequence (SScrofa 11.1). No somatic mutation was found in any coding exon of the 5 genes among three different individuals carrying melanoma lesions, in four tumor samples, representing different stages of interest (not shown).

**Figure 4:**
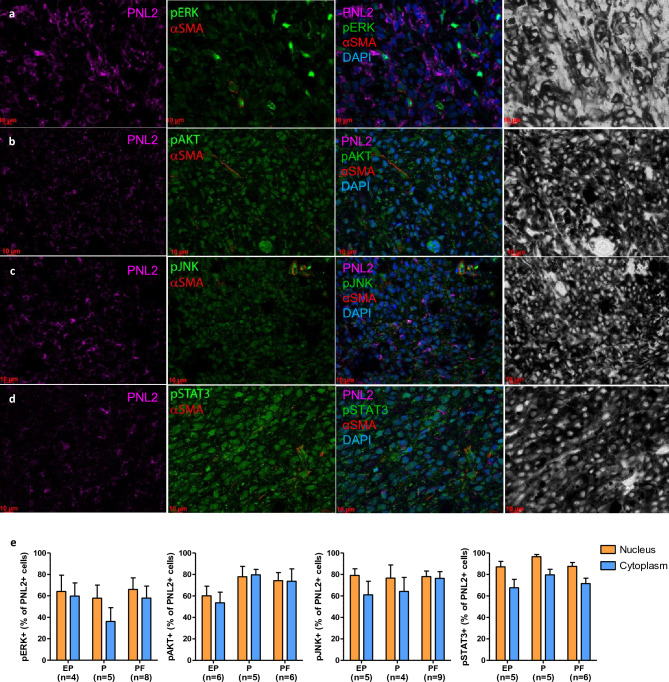
Immunofluorescence analysis of phosphoprotein labeling in MeLiM melanoma. Representative regions of EP lesions immunostained for melanocytic marker PNL2 (purple), pERK (**a**), pAKT (**b**), pJNK (**c**) and pSTAT3 (**d**) (green) and pericyte marker αSMA (red). Third column: merged with DAPI for nuclei (blue). Last column: respective brightfield. Scale bars 10µm. (**e**) Quantification of nuclear and cytoplasmic pERK, pAKT, pJNK and pSTAT3 positive cells among PNL2^+^ cells.

Nonetheless, we examined whether signaling pathways related to tumor growth reported to be active in human melanoma were activated in MeLiM melanomas. Immunofluorescence for phosphorylated ERK, AKT and JNK, effector proteins of the proliferative and stress response MAPK pathways and the survival AKT pathway showed spots of high homogeneous signal for pERK1/2, pAKT and pJNK with cytoplasmic and nuclear distribution (Fig. 4a–c). We also probed the angiogenic pathway with pSTAT3 and αSMA immunofluorescence, which revealed high signal (Fig. 4d). Quantification of the above immunofluorescence data on PNL2^+^ cells at EP, P and PF stages did not differ significantly (Fig. 4e). These results agree with a high proliferation rate of MeLiM tumor cells.

Making use of the RNAseq data, we investigated whether other pathways related to cancer were activated in MeLiM melanomas. As with the previous GO hallmark analysis, we thus performed FSEA for KEGG pathways among expressed genes in at least one of the evolution stage groups or among DE genes between groups (Tables S6 and S7). We found that 10 out of 21 cancer-related KEGG pathways were significantly enriched in expressed or DE genes: p53, mTOR, Notch, VEGF signaling pathways, cell cycle, apoptosis, adherens junction at all stages, while TGF-beta was more specifically expressed at PF stage consistent with fibrosis. In agreement, JAK-STAT signaling pathway and cytokine-cytokine receptor interaction showed the most significantly DE genes between EP and PF stages (Fig. S5).

All in all, despite no evidence of specific oncogenic mutations driving their activation, canonical proliferative, survival and tumorigenesis pathways were activated in MeLiM melanomas during all tumor progression phases, unlike normal skin melanocytes. Simultaneous activation of signalling pathways is a hallmark of malignancy^42^. MAPK pathways are essential for proliferation providing a sustained proliferative signal. BRAF and MEK inhibitors have been used as therapeutic targets for locally advanced unresectable or metastatic *BRAFV600*-mutant melanoma patients for a decade^43^. All MeLiM melanomas showed extensive regions of intense pERK signal on melanoma cells. Activation of MAPK pathways is also known to result in differentiation, survival, angiogenesis and metastasis. However, the absence of mutations in the major human melanoma driver genes makes the MeLiM minipig a model for triple wild-type genomic classification^23^. The MeLiM model could serve as source of alternative mutations in the MAPK pathway^26^.

### A dense infiltration of immune cells occurs during MeLiM melanoma progression

**Figure 5:**
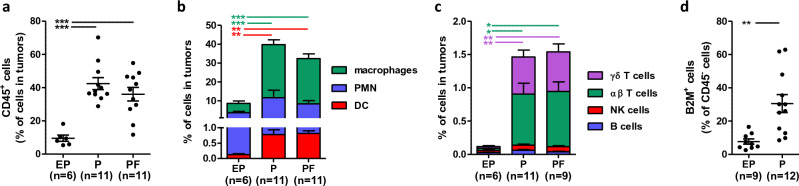
Cytometric analysis of cells in MeLiM tumors. (**a**) Proportion of immune (CD45^+^) cells among total cells in tumors. Proportions of myeloid (**b**) and lymphoid (**c**) cells among total cells in tumors. (**d**) Proportion of B2M^+^ cells among tumor CD45^-^ cells.

As a *proxy* of “tumor promoting inflammation” we quantified microenvironment changes in early melanomas by flow cytometry after tissue dissociation. The ratio of intralesional infiltrating immune cells (CD45^+^) was 9.5% in EP lesions and increased around 40% at later stages (Fig. 5a). The proportion of macrophages surged from 5.0% in EP to a massive 28.1% in the P group and was still predominant in PF (24.0%) in comparison to polymorphonuclear cells (PMN) (7.6%) (Fig. 5b). Interestingly, even if in low proportion, dendritic cells (DC) were also found more abundant at later stages (from 0.1 to 0.8%). Lymphoid cell infiltration was weak, representing 0.1% in EP lesions and increasing to 1.5% in P and PF lesions. More specifically, lymphoid cells infiltrating P and PF lesions were predominantly αβ T cells (0.8%) and γδ T cells (0.6%) (Fig. 5c). These data are in accordance with the enrichment analysis of transcriptomic data: an immune response is set in place during progression and tumors are densely infiltrated by myeloid immune cells and less by lymphoid immune cells. With an initial CD45^+^ infiltration of less than 10% and T lymphocytes almost undetected in the early stages of tumor progression, MeLiM melanomas could be considered cold tumors^44^. However, these rapidly growing nodules without inflammation are swiftly infiltrated, with a massive increase in macrophages, and to a lesser extent DC and T lymphocytes, which continue to evolve during regression^21^. The substantial proportion of MeLiM melanoma cells displaying pJNK signal, a pro-inflammatory marker, could drive this infiltration although it may also promote cell survival^45^.

### Early MeLiM melanoma cells express low B2M

Considering the initially low infiltration, we also examined the expression levels of MHCI through B2M by flow cytometry in tumor cells (CD45^-^). As shown in Fig. 5d, only 7.7 ± 4.7% of CD45^-^ cells expressed B2M in EP group. This proportion increased in the P group (30.5 ± 18.7% of CD45-cells). Of note, in control skin > 90% of fibroblasts are B2M^+^ (data not shown) preventing quantification of B2M in PF lesion melanoma cells. These data suggest an avoidance of immune destruction by MeLiM melanoma cells. One of the mechanisms of relapse after immunotherapies concerns the loss of MHC complexes or down regulation of co-stimulatory molecules. Among the best characterized examples is the loss of the B2M gene^46^. The loss of B2M expression is observed in 40–90% of tumors^47^. It is interesting to point out that in humans, low MHCI expression is associated with invasive and metastatic phenotype and resistance to immune-therapy^48^. We show here that MeLiM tumors express low B2M, an additional aspect of malignancy.

### Successful serial transplantation of MeLiM melanoma xenografts

**Figure 6:**
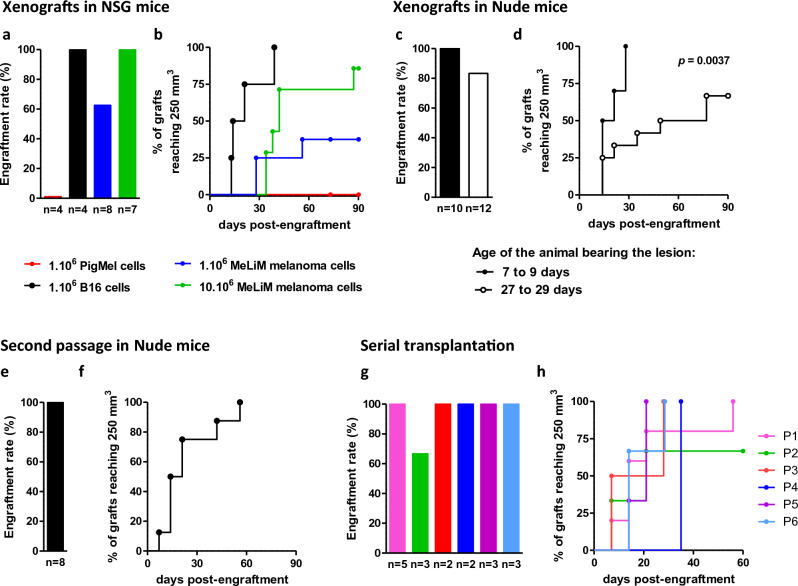
Malignant characteristics of MeLiM melanoma cells in vivo. Analysis of xenograft growth of MeLiM melanoma cells into immunocompromised mice. Engraftment rate (**a**) and longitudinal development (**b**) of subcutaneous nodules reaching 250 mm^3^ at the site of injection of MeLiM melanoma cells at 1 million (blue) and 10 million (green) in NSG mice. For comparison, murine melanoma (B16, black) and porcine non-transformed melanocytic (*PigMel*, red) cell lines were injected. Engraftment rate (**c**) and nodule growth (**d**) of MeLiM melanoma cells from piglets aged 7–9 days (black circle) and 27–29 days (open circle) into nude mice. Growth of reimplanted MeLiM melanoma nodules in nude mice from lesions harvested from piglets aged 39–146 days (**e**, **f**). Melanoma cells could be transplanted up to 7 passages as shown for a piglet melanoma cells (**g**, **h**).

To determine whether MeLiM melanoma cells were malignant in vivo, activating invasion and metastasis, we tested their ability to engraft into immunocompromised mice. For that purpose, 1–10 million cells isolated from dissociated lesions excised from 3 to 14-day-old MeLiM piglets were injected subcutaneously in severely immunocompromised NSG mice. Engraftment of 1 million MeLiM melanoma cells from 4 pigs formed nodules in 62% of NSG mice (5 out of 8 mice), while the same amount of highly tumorigenic murine melanoma B16 control cells engrafted in 100% of mice (n = 4), unlike non-tumorigenic porcine *PigMel* cells which formed no nodule (n = 4) (Fig. 6a). Xenotransplation of 10 million of MeLiM melanoma cells had a tumor formation rate of 100% (n = 7) in NSG mice. Although 37.5% of mice injected with 1 million MeLiM melanoma cells displayed a nodule reaching 250 mm^3^, the proportion increased to 85.7% in a median time of 42 days for mice receiving 10 million cells (Fig. 6b).

In order to follow the metastasis process, engraftment was performed into hairless nude mice*.* When melanoma cells came from lesions in early progression (EP group) sampled on 2 piglets aged 7 and 9 days, 100% of engrafted mice (n = 4 and 6, respectively) developed a conspicuous pigmented tumor mass (Fig. 6c). All nodules reached 250 mm^3^ in a median time of 17.5 days (Fig. 6d). When melanoma cells came from lesions with partial regression (PF group) sampled on 3 piglets aged 27 and 29 days, engraftment (in 5, 4 and 3 mice, respectively) was successful in 83.3% of mice (10 out of 12, Fig. 6c) and nodules reached 250 mm^3^ in 66.7% (8 out of 12) in a median time of 63 days (Fig. 6d). These results support that PF lesions harbor malignant cells while suggest a difference in endogenous proliferative potential of MeLiM melanoma cells between these evolution stages (p = 0.0037). Of note, pigmented lymph nodes, a sign of metastasis, were observed in nearly all cases (Fig. S6a).

A malignant population of tumor cells is not only capable of primary engraftment (direct from the pig lesion), but also of serial transplantation. We then tested whether MeLiM melanoma cells could be re-engrafted and whether older animal’s cells would lose tumorigenesis capacity. At the first xenograft passage (X1), mice engrafted with xenografts collected from 3 lesions sampled after 39 to 146 days of growth in mice was successful in 100% of cases (n = 8, Fig. 6e), reaching 250 mm^3^ in 100% of mice in a median time of 17.5 days (Fig. 6f). Pigmented lymph nodes were also observed in all engrafted mice (Fig. S6b). Moreover, one tumor has been serially engrafted 7 times in different mice, each time with a high rate of engraftment (Fig. 6g,h) and with pigmented lymph nodes observed in all mice harboring nodules (Fig. S6c). In total this initial tumor was kept in progression during 500 days. Histologic and immunofluorescence analysis of tumor cell proliferation and related pathway markers confirmed the presence of MITF^+^ Ki67^+^cells, and pERK^+^ and pAKT^+^ cells in PNL2^+^ cells (Fig. S7). Tumor grafts resembled the original tumor from which they derived. High in vivo serial transplantation engraftment rates with metastasis, phosphoprotein activation, histopathology and clinical features similar to the original tumor point to what is defined as a malignant phenotype.

These results suggest that MeLiM melanoma cells proliferate unrestrictedly in the absence of an immune response in immunocompromised mice. Xenografts in immunocompromised mice address the potential of cancer cells to proliferate extensively and to form tumors without the bias of adapting to tissue culture conditions. We have shown that orthotopically injected MeLiM tumors are able to grow in immunocompromised mice and even be serially transplanted for numerous passages without loss of proliferation rate, despite the fact that melanomas regress in 95% of cases in our experimental unit. The mice also showed pigmented adenomegaly, suggesting invasion and migration of cancer cells to distant organs. The rate of transplantability of MeLiM melanoma is comparable to that in humans. Quintana et al.^49^, reported that 17 out of 19 patient melanomas grew in immunocompromised mice and could almost always be serially passaged. Growth after serial xenotransplantation addresses the enabling of replicative immortality hallmark of MeLiM melanomas.

MeLiM develop multiple melanomas that spontaneously regress. However, our data show that despite their commitment to regress, MeLiM melanoma cells harbor conspicuous autonomous malignancy traits all along. Accordingly, proliferative, survival and angiogenic signalling pathways are activated. Functionally, we show that MeLiM melanoma cells are capable to grow in immunocompromised mice, with serial passages and for a longer time than in MeLiM pigs. Interestingly, evolutive features during tumor progression concern a slight loss in xenograft potential with age while all other measured features remain constant, with the notable exception of the immune infiltration that gradually then steeply increases. We conclude that MeLiM melanomas share several malignancy characteristics with human melanomas.

Interestingly, a recent study reported that histopathological regression was observed in 10% of melanoma patients with positive sentinel lymph node biopsy and had significant correlation with relapse free survival^50^. Finally, our data on MeLiM melanomas reveal several malignancy traits. The combination of these features with the successful spontaneous regression of these tumors make it an outstanding model of an efficient anti-tumor immune response. Further studies are required to describe the mechanisms of regression in this minipig model.

## Methods

### Pigs, tumor clinical examination and sampling

MeLiM minipigs were maintained in a certified swine facility according to European guidelines. Animal experimentation protocols approved by the Institutional Animal Care and Use Committee (CEEA45) were approved by the French Ministry of Research (MESRI n° 2016022414473586, 2021062410172950). Authors complied with ARRIVE guidelines. MeLiM pigs (28 males, 34 females, Table S1) from 32 litters were examined weekly until 50 days of age for the presence of cutaneous melanoma. Clinical examination consisted of identification of new cutaneous lesions and palpable adenomegaly, and measurement of evolution of all identified lesions using calipers. Phenotypic data reported in Table S2 include lesion count at birth, number of lesions per individual, palpable adenomegaly with pig age at onset. For each excised lesion, shape, diameter, ulceration, pigmentation and pig age were recorded. Tumors were surgically excised under isoflurane gas anesthesia and intra-muscular meloxicam. Tumor samples were divided into 3 parts and stored in liquid nitrogen, in 4% paraformaldehyde for 48h or in solution A (PBS containing 5% Vetedine solution (Vetoquinol), 200 units/mL penicillin, 200 µg/mL streptomycin (all from Gibco)) for dissociation. Table S2 also summarizes the use of each lesion in the current study.

### Histopathology and Immunofluorescence labeling

Hematoxylin–eosin-saffron (HES)-stained paraffin-embedded sections stained were digitally scanned (Pannoramic SCAN, 3DHISTECH) with a 20× objective and visualized using CaseViewer (3DHISTECH). Histologic assessment and skin extension were graded according to human classification using Clark levels^51^. Lesion cross-sectional size parameters were evaluated with ulceration, histologic profile, vascularity, lymphoid cell infiltration. Regression was characterized by the presence of dermal fibrosis. Immunofluorescence was performed with mouse anti-PNL2 (1:150, Santa Cruz Biotechnologies), anti-αSMA (1:200, Dako), anti-MITF (1:100, Zymed), rabbit polyclonal anti-pERK(Thr202/Tyr204), anti-pJNK(Thr183/Tyr185), anti-pSTAT3(Tyr705) and anti-pAKT (Ser473) (1:200, Cell Signaling) and rabbit polyclonal anti-Ki67 (1:1000, Abcam) antibodies. Nuclear counterstaining was performed with 4ʹ,6ʹ-diamidino-2-phenylindole (DAPI) (1:1000, Invitrogen). Sections were examined using a Zeiss Axio Observer Z1M ApoTome microscope (Carl Zeiss). Controls without the first antibodies showed no unspecific labeling. Images were processed using the *AxioVision* computer program version 4.6 (Carl Zeiss). Figures are representative of the skin samples evaluated. All images shown are individual sections from the z-series stack. Final figures were assembled using Adobe Photoshop CS3 (Adobe Systems; USA). Quantification of Ki67 and p-kinase positive nuclei was performed using Fiji and R.

### Preparation of cell suspension from excised tumors

Cell suspensions were prepared by removing the epidermis and hypodermis, decontaminating the tissues for 30 min in solution A, 0.2% Fungizone (Gibco). Samples were weighed, minced, and enzymatically treated^52^ with 4 mg/mL collagenase B, 0.1 mg/mL DNase I (Roche) in DMEM containing 100 units/mL penicillin, 100 µg/mL streptomycin, 2mM L-glutamine, 0.5mM EDTA, and 2% FBS (all from Gibco) twice 20 min at 37 °C with shaking. Intensive washes were performed in the same medium without enzymes to remove released melanin. Absolute cell number and viability were determined using the ViaCount assay on an easyCyte 6HT-2L Guava flow cytometer (Millipore) according to the manufacturer’s instructions.

### Mice transplants

NOD.Cg-*Prkdc*^*scid*^*Il2rg*^*tm1Wjll*^/SzJ (NSG) immunodeficient mice from Charles River Laboratories and Rj:NMRI-*Foxn1*^*nu/nu*^ nude mice from Janvier Labs were maintained under specific pathogen-free conditions in a certified animal facility according to European guidelines. Experimental procedures were performed in accordance with French ethics committee (CEEA26) and MESRI approval (n° 2019010816113920). Authors complied with ARRIVE guidelines. Mouse experiments were performed on 6 to 8 weeks old males. Dissociated piglet tumor cells obtained as described above were washed with PBS to remove FCS and 0.1 mL (containing 1.10^6^ to 10.10^6^ tumor cells) was injected subcutaneously into the ventral lower right side of a mouse under isoflurane gas anesthesia. B16 murine melanoma cells^18^ and *PigMel* porcine melanocyte cells^53^ were injected as controls. Tumor volume was monitored weekly using calipers and the formula *V* = 0.5 × length × width^2^, with the longest diameter as length and its perpendicular as width. Mice with tumors that reached 4000 mm^3^, exhibited ulceration or showed signs of declining health were euthanized by cervical dislocation. Tumor formation was monitored for a maximum of 5 months after transplantation before mice were euthanized. At sacrifice, nodules were excised, one part was fixed in 4% paraformaldehyde the other part was used to prepare a cell suspension as above for serial transplantation. Complete necropsy and systematic pathologic analysis were performed on all mice as described for pigs. Lymphadenopathies were monitored in nude mice by observation of pigmentation through the skin at lymph node sites.

### RNAseq

Total RNA from 4 frozen tumors of each evolution stage (EP, P and PF) was extracted from 50 × 16 µm cryostat sections (CryoStar NX50) using the NucleoSpin RNA-XS extraction kit (Macherey Nagel). RNA quality was assessed using the Agilent eukaryote total RNA 6000 Nano Kit (Agilent 2100 BioAnalyzer System, RIN 7.4–9.9, average 8.9). The amount of RNA was determined using the Nanodrop (Thermo Fischer Scientific). Libraries were prepared from 500 ng of RNA using Roche kit and sequenced for 100 bp paired-end on the Illumina NovaSeq 6000 system using Novaseq 6000SP Reagent Kit (200 cycles), 500 million reads, on the iGenSeq transcriptomic platform at the Brain and Spine Institute (ICM, Paris, France). After removing adapter sequences from raw RNAseq reads, gene expression was performed by transcript pseudo-alignment with *kallisto*^54^ using the porcine reference genome annotation available at Ensembl 104 (www.ensembl.org)*.* The 21,639 genes with non-zero total read counts were retained for MDS analysis after TMM normalization. For gene expression analysis at each evolution stage group, a threshold of TPM > 1 in 3 of the 4 tumors was set. Differential analysis of gene level abundances was performed using *DESeq2*^55^. A negative binomial generalized linear model was fitted with fixed effects to control for sex and evolution stage group (EP, P, PF). For each pairwise comparison between evolution stage groups, a significance threshold of α = 5% (Benjamini–Hochberg corrected *p*-values) was used to identify DE genes. Gene names were annotated using the Biomart and HCOP ortholog databases^50^ (https://www.genenames.org/tools/hcop/, downloaded 03/17/2022). FSEAs were performed using the tmod (v0.50.11)^56^ R package. Hypergeometric tests were used to determine enrichment of a foreground set of genes (expressed genes in each group) in the background set of 21,639 genes. The Coincident Extreme Ranks in Numerical Observations (CERNO) method was used to identify gene enrichment in ranked lists according to the *p*-value from DE analyses. Gene Ontology Biological Process terms (msigdbr_7.5.1)^57^ and human KEGG pathways (KEGGREST 1.38.0) were used for gene set interpretation. The list of GO terms assigned to cancer hallmarks was extracted from the Chen et al. (Table S11 of Ref.^6^). The list of KEGG pathways related to cancer was extracted from the hsa05200 “pathways in cancer” map^58^ (Table S8).

### Cancer genes sequencing

cDNA sequencing was performed on 4 snap-frozen tumors sampled at different times from 3 MeLiM animals. Briefly, total RNA was extracted from tumor slices with the Nucleospin RNA minikit (Macherey–Nagel). RNA quality and quantity were evaluated by Bioanalyzer and Nanodrop instruments, respectively. cDNA was synthetized with Superscript III synthesis kit (ThermoFisher), with 1 µg of RNA. PCR products were amplified by using HotStarTaq DNA polymerase according to the manufacturer’s instructions (Qiagen), with 0.5 µM primers and 5 ng cDNA. Primer sequences are summarized in Table S9. Sanger sequencing of amplicons was outsourced.

### Custom exome-based sequencing

Custom-designed exome capture arrays were used as a platform to study genomic variation in MeLiM tumors. Briefly, in another project, 9 genomic regions were selected based on significant association signals with melanoma incidence and metastasis in the MeLiM model^16^. Within these loci, we listed features annotated as exons or ESTs, retrieved their genomic coordinates, and added 1000 bp on each side of the features (Sscrofa9.2). Nimblegen then performed repeat masking, small features extension and probe design, resulting in a 385 K probe array targeting a total of 6.56 Mb of genomic DNA. DNA was extracted from multiple tumor tissues from 3 different animals (Table S10). Matching normal blood lymphocytes DNA was extracted for each animal. Library preparation was performed using TruSeq® DNA Library Prep Kit (Illumina®) according to the manufacturer’s instructions. DNA libraries were further hybridized to Nimblegen capture arrays according to the Nimblegen protocol. DNA was eluted from arrays and PCR amplified prior to sequencing at the Centre National de Recherche en Génomique Humaine (CNRGH). Paired-end reads were aligned to the porcine reference genome (Sscrofa10.2) using BWA (version 0.6.1-R104). Reads were further processed using Picard-Tools (version 1.119). Somatic variants were detected using the Mutect2 tool^59^ by comparing tumor and normal DNA sequences aligned to the reference genome. The generated VCF files were further used for SNV and indel counting in the different samples. SNVs were also annotated using Variant Effect Predictor (v84).

### Flow cytometry analyses

Tumor cell suspensions were stained as previously described for blood cell analysis^60^. Briefly, two antibody combinations were used with the aqua LIVE/DEAD® Fixable Dead Cell Staining Kit (Thermo Fisher Scientific) for viability, to identify lymphoid cells (Combination A: CD45, CD3, CD8α, γδTCR, and CD79a) and myeloid cells (Combination B: CD45, MHC II, CD172a, and PG68A) as previously described. In combination B, a biotinylated mouse anti-Beta-2-microglobulin monoclonal antibody (Clone B2M-02, 2 µg/mL, Origene) was also added and revealed with streptavidin-BV786 (0.1 µg/mL, BD Biosciences). If necessary, intracellular staining was performed with Foxp3/Transcription Factor Staining Buffer Set (eBioscience). Five million cells were processed in each combination and finally fixed in BD CellFIX solution before analysis on a BD LSR Fortessa cytometer (BD Biosciences). Data were analyzed using FlowJo V10 software.

### Supplementary Information

Supplementary Figures.

Supplementary Tables.

## Data Availability

Raw RNA sequencing data are deposited at the SRA (numberPRJNA997124).
