# Supplementary material for: Malignant features of minipig melanomas prior to spontaneous regression
Source: Sci Rep. 2024 Apr 22;14:9240. doi: 10.1038/s41598-024-59741-w (PMC11035550; doi:10.1038/s41598-024-59741-w)
Supplement: Supplementary file 1 — Supplementary Figures. [file 41598_2024_59741_MOESM1_ESM.pdf]

Supplementary Figures

MALIGNANT FEATURES OF SPONTANEOUSLY REGRESSING MINIPIG MELANOMAS

Héloïse Débare[1], Fany Blanc[1], Guillaume Piton[1,2,3], Jean-Jacques Leplat[1,2], Silvia Vincent-Naulleau[1,2], Julie Rivière[1], Marthe Vilotte[1], Sylvain Marthey[1], Jérôme Lecardonnel[1], Jean-Luc Coville[1], Jordi Estellé[1], Andrea Rau[1], Emmanuelle Bourneuf[1,2,3], Giorgia Egidy[1*]

[1] Université Paris-Saclay, INRAE, AgroParisTech, GABI, 78350 Jouy-en-Josas, France; [2]Université Paris-Saclay, CEA, Stabilité Génétique Cellules Souches et Radiations, F-92260 Fontenay-aux-Roses, France ; [3]Université de Paris Cité, CEA, Stabilité Génétique Cellules Souches et Radiations, F-92260 Fontenay-aux-Roses, France

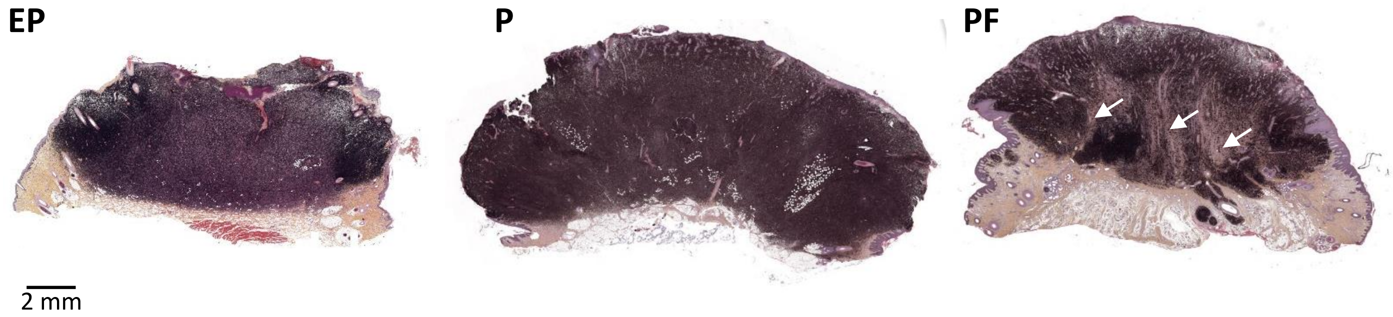

Figure S1: Histology of MeLiM melanoma lesions. HES staining of lesions from groups EP, P and PF. One representative tumor from each group is shown. White arrows indicate zones of dermal fibrosis, histologic criteria of partial regression.

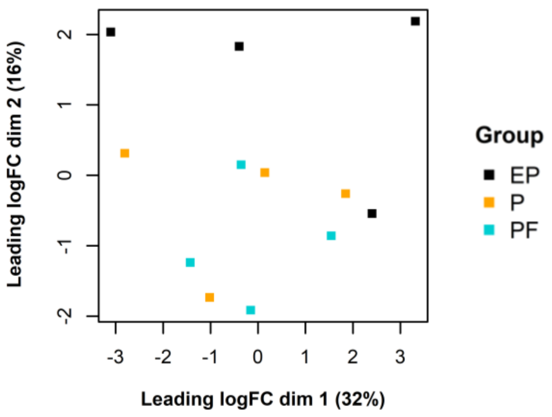

Figure S2: MDS plot of RNA-seq expression profiles in two dimensions. The 21639 genes with non-zero total read counts were retained for MDS analysis after TMM normalization. Samples are colored according to their group.

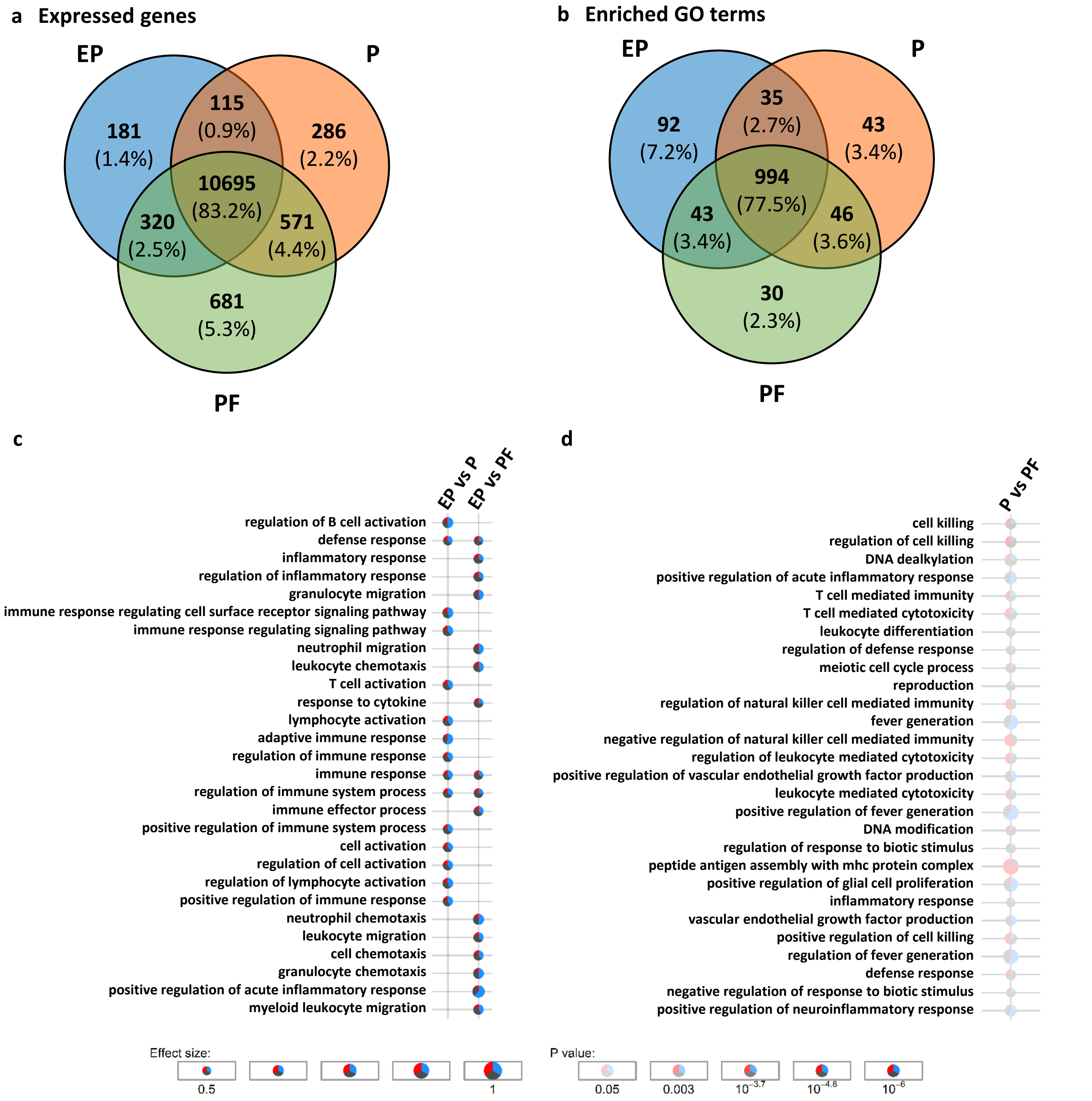

Figure S3: (a) Venn diagram of expressed genes in EP, P and PF groups (b) Venn diagram of GO terms found enriched by a Feature Set Enrichment Analysis (FSEA) in expressed genes in EP, P and PF groups. Hypergeometric tests were used to determine the enrichment of expressed genes in each group (foreground set) among the total RNAseq gene set (background set corresponding to 16559 genes). **(c-d) FSEA to evaluate enriched GO terms in differentially expressed (DE) genes comparing EP vs P and EP vs PF (c), and P vs PF (d) groups.** The CERNO method was used to analyze gene enrichment in ranked lists of p-values from DE analyses. The transparency of color indicates the strength of the p-value; the effect size (E or AUC) is indicated by the plot size. Only the most significant GO terms are represented in (a) (adjusted p-value < 10^{-6}). Significantly ($p < 0.05$) up-regulated genes in the first term of the comparison are colored red while downregulated genes are colored blue; others are colored gray.

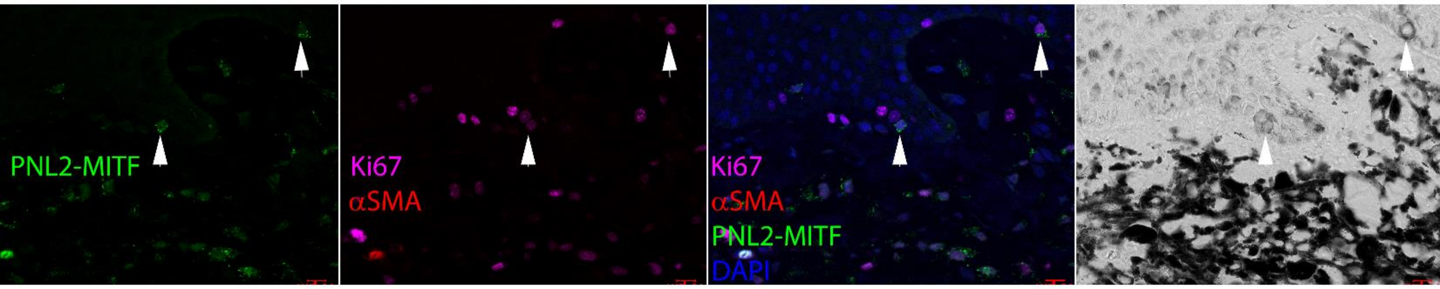

Figure S4: Ki67 immunostaining of skin adjacent to melanoma. Representative region of skin overlying a melanoma of a 22-day-old pig with staining for the melanocytic marker PNL2 (cytoplasm) and MITF (nuclear) (purple, arrowheads), proliferation marker Ki67 (green) and pericyte marker αSMA (red). Third column: merge of the previous with DAPI for nuclei (blue). Last column: brightfield with high melanin content. Scale bars 10µm.

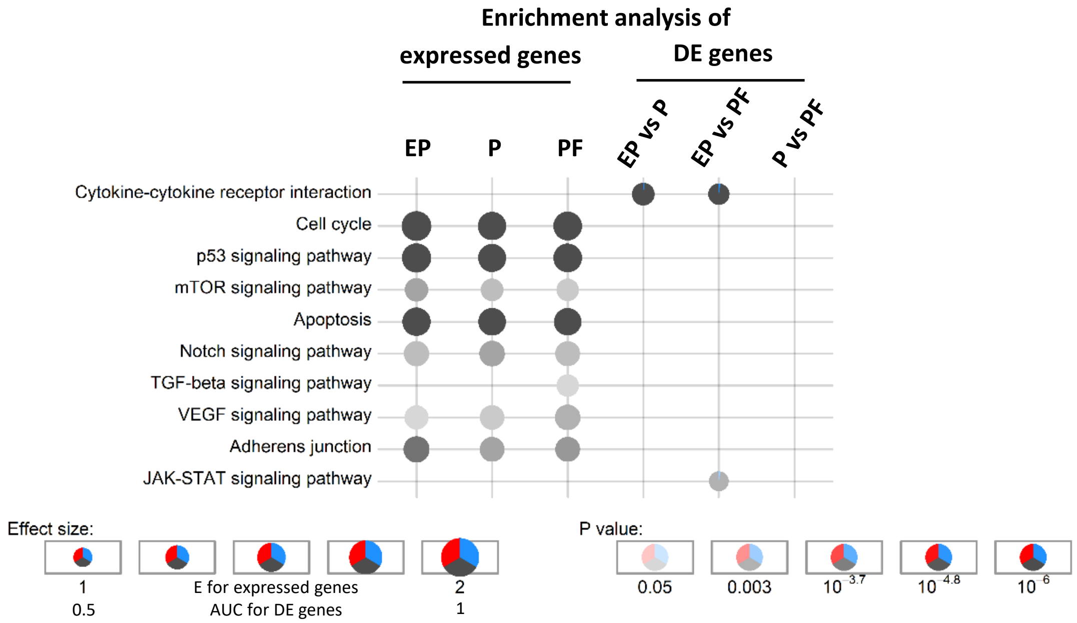

Figure S5: Cancer-related KEGG pathways enriched in MeLiM tumors. Longitudinal transcriptomic analysis and FSEA in KEGG pathways of genes expressed in EP, P and PF groups and in DE genes between groups. Hypergeometric tests were used to determine the enrichment of expressed genes in each group (foreground set) among the total RNAseq gene set (background set corresponding to 16482 genes). The Coincident Extreme Ranks in Numerical Observations (CERNO) method was used to analyze gene enrichment in ranked lists of the *p*-value of the DE analyses. Only enriched KEGG pathways related to cancer were plotted. The strength of the *p*-value is indicated by the transparency of the color; the effect size (E or AUC) is indicated by the plot size. Significantly ($p < 0.05$) downregulated DE genes are colored blue; others are colored gray.

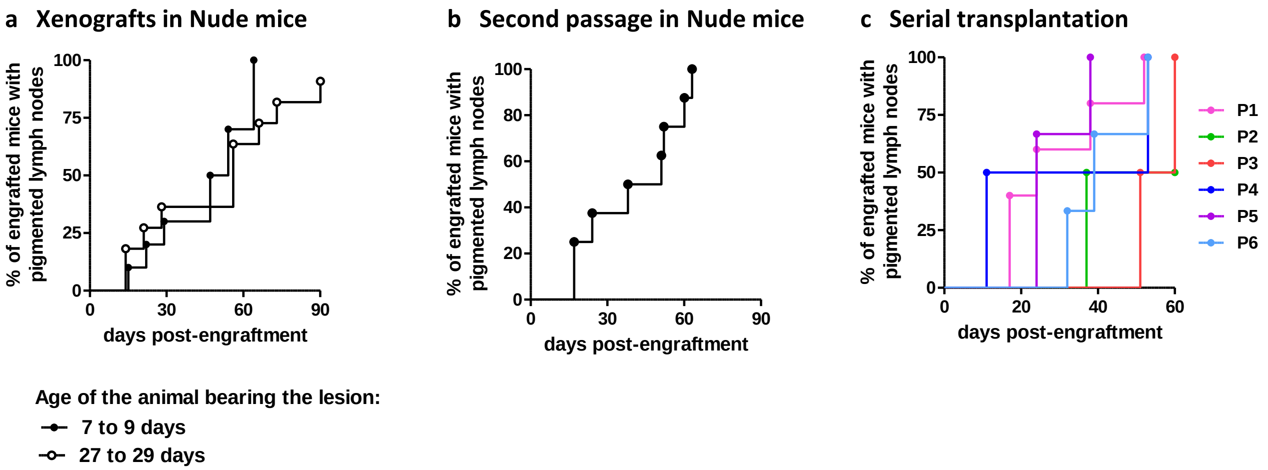

Figure S6: Observation of pigmented lymph nodes in nude mice, a sign of metastasis. (a) Percentage of nude mice with pigmented lymph nodes on primary xenografts of lesions from animals aged 7-9 days (•), or 27-29 days (º), (b) on the second passage of engraftment, (c) on serial transplantation of an 8-day-old lesion.

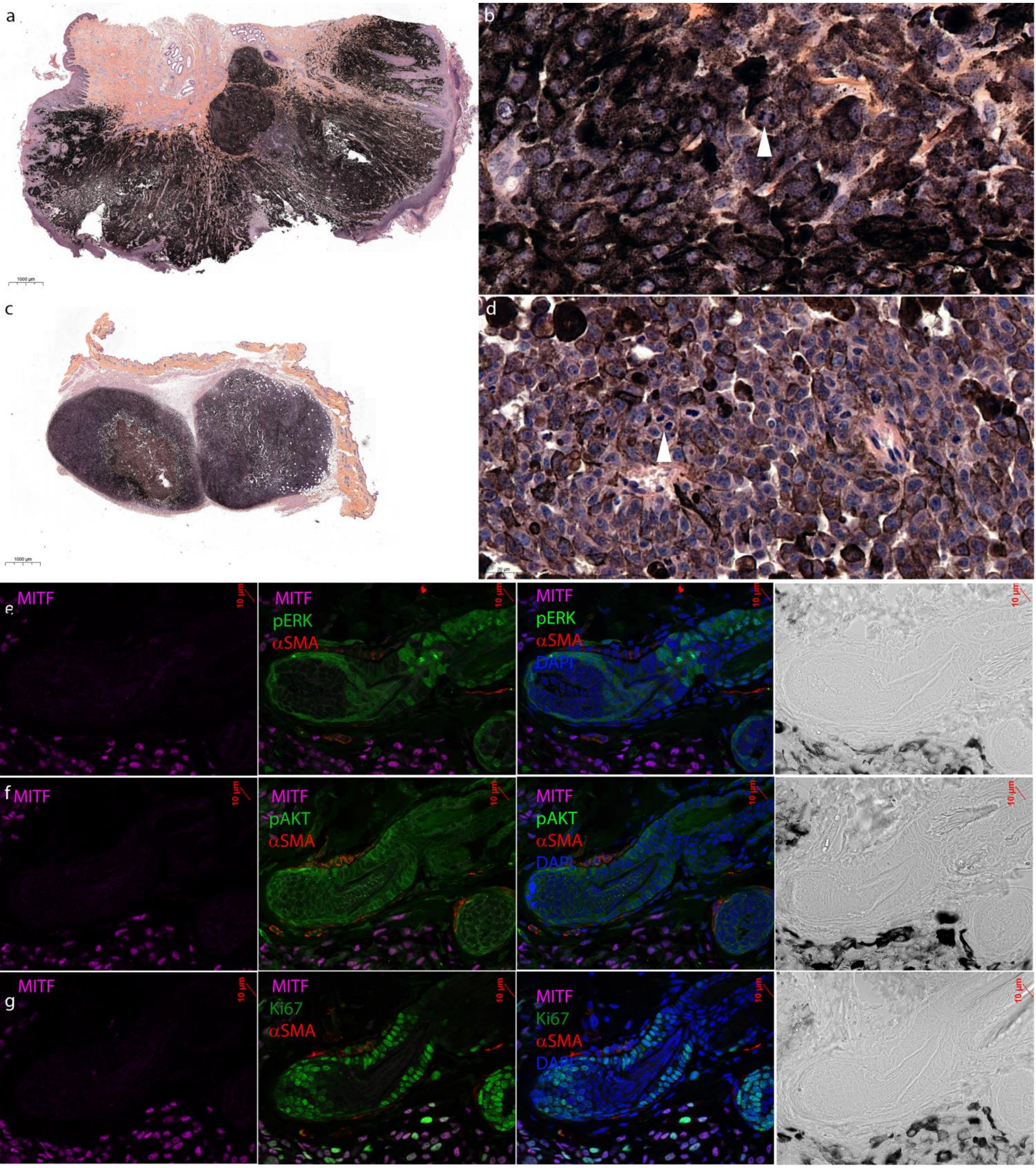

Figure S7: Histologic characteristics of MeLiM melanomas transplanted into nude mice. Hematoxilin-eosin-safran (HES) staining of a MeLiM melanoma grown in nude mouse (c-d) compared to its original tissue (a-b). Representative regions of grown engrafted tumor with antibody staining for MITF and aSMA co-labeled with pERK (e), pAKT (f) and Ki67 (g). Scale bars 1cm (a, c), 20µm (b, d), 10µm (e-g).
